# Supplementary material for: The Influence of Maternal Vitamin E Concentrations in Different Trimesters on Gestational Diabetes and Large-for-Gestational-Age: A Retrospective Study in China
Source: Nutrients. 2022 Apr 14;14(8):1629. doi: 10.3390/nu14081629 (PMC9032640; doi:10.3390/nu14081629)
Supplement: Supplementary file 1 [file nutrients-14-01629-s001.zip › nutrients-1643824-supplementary.pdf]

nutrients MDPI

Supplementary materials

Supplementary Table S1. The association between maternal characteristics and GDM, by univariate analyses (n=16,107).

Variables	All participants N	GDM n (%)	Not GDM n (%)	P [a]
**Maternal age**				
≤20	110	17(15.5)	93(84.5)	**<0.001**
21-30	10548	2640(25.0)	7908(75.0)	
>30	5449	1845(33.9)	3604(66.1)	
**Parity**				
Primiparous	9863	2623(26.6)	7240(73.4)	**<0.001**
Multiparous	6244	1879(30.1)	4365(69.9)	
**Maternal ethnicity**				
Han	15149	4241(28.0)	10908(72.0)	0.629
Ethnic minorities	957	261(27.3)	696(72.7)	
**Maternal education**				
Below high school	3389	993(29.3)	2396(70.7)	0.479
High school or college	6249	1758(28.1)	4491(71.9)	
University or higher	6079	1735(28.5)	4344(71.5)	
**Maternal pre-pregnancy BMI**				
<18.50	1630	256(15.7)	1374(84.3)	**<0.001**
18.5-23.99	9766	2480(25.4)	7286(74.6)	
24.00-27.99	3237	1244(38.4)	1993(61.6)	
≥28.00	1093	507(46.4)	586(53.6)	
**Folic acid usage**				
Yes	14722	4144(28.1)	10578(71.9)	**0.068**
No	1385	358(25.8)	1027(74.2)	

a: The p value is reported from Chi-square test.

Supplementary Table S2. The association between maternal characteristics and large for gestational age (LGA), by univariate analyses (n=18,699)

Variables	All participants N	LGA n (%)	Non-LGA n (%)	P [a]
**Maternal age**				
≤20	127	21(16.5)	106(83.5)	**<0.001**
21-30	12059	1869(15.5)	10190(84.5)	
>30	6513	1429(21.9)	5084(78.1)	
**Parity**				
Primiparous	11139	1681(15.1)	9458(84.9)	**<0.001**
Multiparous	7560	1638(21.7)	5922(78.3)	
**Maternal ethnicity**				
Han	17578	3117(17.7)	14461(82.3)	0.797
Ethnic minorities	1120	202(18.0)	918(82.0)	
**Maternal education**				
Below high school	3915	776(19.8)	3139(80.2)	**<0.001**
High school or college	7257	1247(17.2)	6010(82.8)	
University or higher	7142	1212(17.0)	5930(83.0)	
**Maternal pre-pregnancy BMI**				
<18.50	1985	156(7.9)	1829(92.1)	**<0.001**
18.5-23.99	11565	1917(16.6)	9648(83.4)	
24.00-27.99	3592	838(23.3)	2754(76.7)	
≥28.00	1189	329(27.7)	860(72.3)	
**Gestational weight gain**				
<10 kg	2186	265(12.1)	1921(87.9)	**<0.001**
10-17 kg	7990	1205(15.1)	6785(84.9)	
> 17 kg	4806	1081(22.5)	3725(77.5)	
**Preterm birth**				
Yes	656	104(15.9)	552(84.1)	0.196
No	18043	3215(17.8)	14828(82.2)	
**Newborn gender**				
Boy	9626	1743(18.1)	7883(81.9)	0.187
Girl	9073	1576(17.4)	7497(82.6)	
**Folic acid usage**				
Yes	17052	3020(17.7)	14032(82.3)	0.653
No	1647	299(18.2)	1348(81.8)	

a: The p value is reported from Chi-square test.

(a)

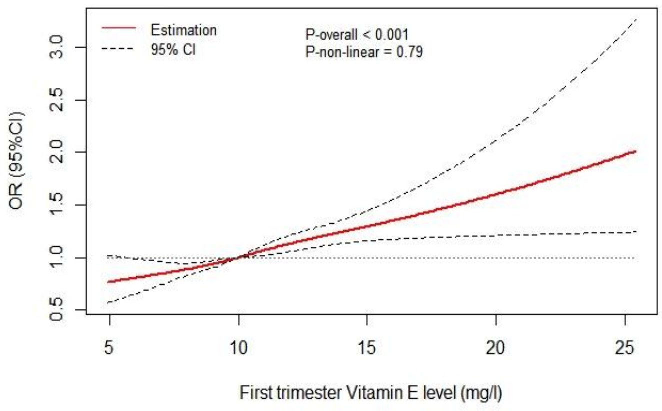

(b)

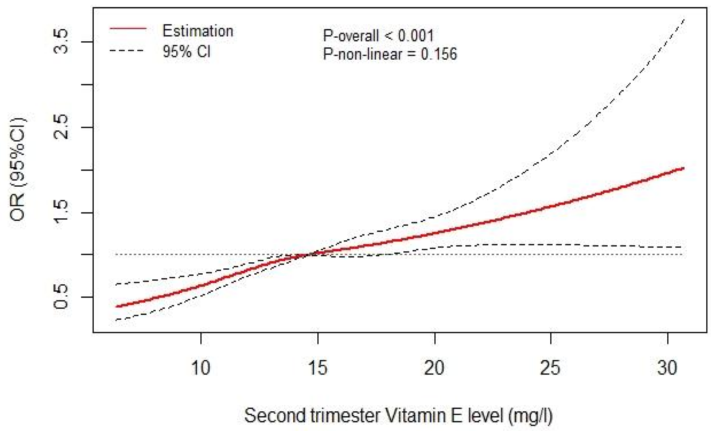

Supplementary Figure S1. The relationship of (a) first trimester and (b) second trimester vitamin E levels and GDM (reference is 20.0 ml/l). Maternal age, parity, maternal pre-pregnancy BMI, folic acid usage were adjusted.

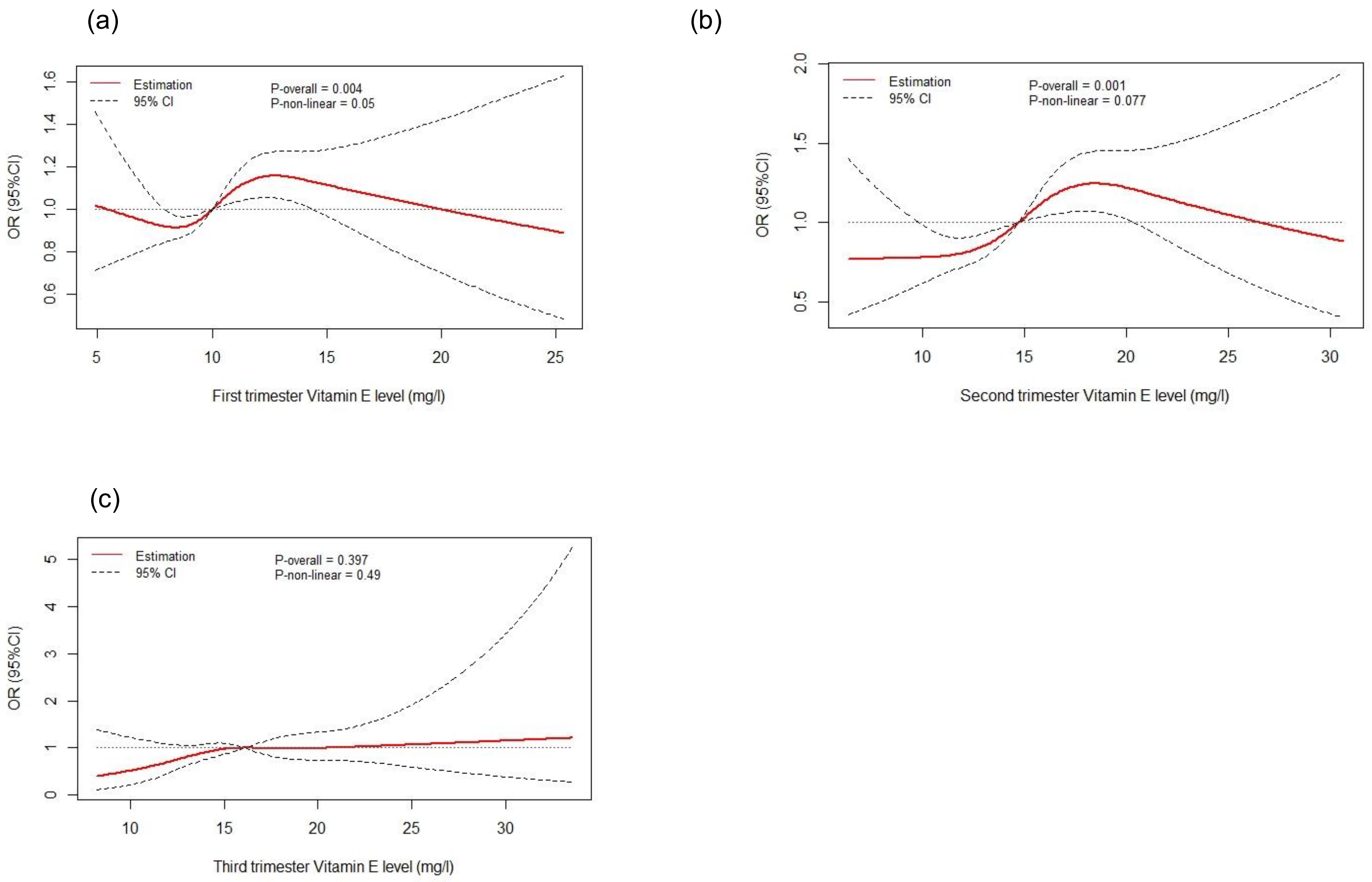

Supplementary Figure S2. The relationship of (a) first trimester, (b) second trimester and (c) third trimester vitamin E levels and LGA (reference is 20.0 ml/l). Maternal age, parity, maternal education, maternal pre-pregnancy BMI and gestational weight gain were adjusted.
